# Ventriculoperitoneal Shunting Versus Endoscopic Third Ventriculostomy for the Surgical Management of Idiopathic Normal Pressure Hydrocephalus: A Retrospective Cohort Analysis

**DOI:** 10.7759/cureus.78347

**Published:** 2025-02-01

**Authors:** Thaddeus D Harbaugh, Mason T Stoltzfus, David R Hallan, Lekhaj Daggubati, Elias B Rizk

**Affiliations:** 1 Neurosurgery, Penn State College of Medicine, Hershey, USA; 2 Neurosurgery, Penn State Health Milton S. Hershey Medical Center, Hershey, USA; 3 Neurosurgery, George Washington University Hospital, Washington, DC, USA

**Keywords:** endoscopic third ventriculostomy, endoscopic third ventriculostomy complications/malfunction, normal-pressure hydrocephalus, ventriculoperitoneal shunt complications/malfunction, ventriculoperitoneal (vp) shunt

## Abstract

Objective

Idiopathic normal pressure hydrocephalus (iNPH) is a chronic condition characterized by ventricular enlargement in the setting of normal opening pressure and presents with gait ataxia, dementia, and urinary incontinence. Surgical treatment options have been shown to be effective for the treatment of iNPH, with the two most common being the placement of a ventriculoperitoneal shunt (VPS) or an endoscopic third ventriculostomy (ETV) procedure. Several studies have compared clinical outcomes across these two modalities but with conflicting results. While some studies report greater symptom resolution and fewer complications with one of the two procedures, others report the opposite. Here, we leveraged a large database to retrospectively compare the clinical outcomes of patients with iNPH who were treated with either VPS or ETV, aiming to provide further insight into this ongoing debate.

Methods

The electronic health record database, TriNetX, was used to isolate patients aged 50 years or older who were diagnosed with iNPH. Patients were then separated into two cohorts by surgical treatment with either VPS or ETV and propensity score matched based on various demographics and comorbidities, yielding 118 matched patients in each cohort for the final outcome analysis. Outcomes were evaluated within 10 years following surgery and included death, subdural hematoma, seizures, falls, urinary incontinence, emergency department visits, CT of the head, and MRI of the brain.

Results

Patients in the VPS cohort were significantly more likely to visit the emergency department (p = 0.0333) and receive a CT scan of the head (p = 0.0001), while patients in the ETV cohort were significantly more likely to receive an MRI of the brain (p < 0.0001). There was no significant difference between the two cohorts in the remaining outcomes, including death (p = 0.8510), subdural hematoma (p = 0.8520), seizures (p = 0.9272), falls (p = 0.0829), and urinary incontinence (p = 0.2902).

Conclusions

Our results showed few differences in outcomes between iNPH patients treated with either VPS or ETV. The differences observed in the use of imaging modalities are not surprising given the nature of each approach, with CT offering efficient visualization of the implanted device in VPS patients and MRI facilitating evaluation of the stoma created during the ETV procedure. Furthermore, the difference in emergency department visits could not be reliably linked to procedural complications as the reason for the visit was unknown. Overall, our findings suggest that outcomes are comparable between these two surgical approaches, though further research is needed to validate this conclusion.

## Introduction

Idiopathic normal pressure hydrocephalus (iNPH) is a chronic disease first described by Hakim et al. in 1965 as a communicating hydrocephalus defined by enlarged ventricles in the setting of a normal lumbar puncture opening pressure and with no obstruction of the craniospinal cerebrospinal fluid (CSF) space [1]. While the pathophysiology of iNPH is not yet fully understood, various explanations have been proposed, including decreased compliance of the subarachnoid space, increased resistance to CSF reabsorption by the cerebral venous system, or benign external hydrocephalus beginning in infancy [2-4]. The symptomatology of iNPH is characterized by the Hakim-Adams clinical triad of gait ataxia, dementia, and urinary incontinence [1]. The incidence of iNPH is approximately 1.58 patients per 100,000 with an average age of onset of approximately 70 years, with no apparent difference in incidence rates between males and females [5,6].

With treatment, some symptoms of iNPH are reversible, making it one of the few treatable causes of dementia [7]. While ventriculoperitoneal shunt (VPS) placement has remained the primary surgical mode of treatment for iNPH, others have suggested the use of endoscopic third ventriculostomy (ETV), given the potential complications associated with VPS surgery [8]. An ETV procedure involves the creation of an opening in the floor of the third ventricle, which allows for CSF outflow into the subarachnoid space, thereby decreasing intraventricular pulse pressure. A VPS functions to divert CSF from the ventricles of the brain to the peritoneal cavity, maintaining physiologic intracranial pressures via valves that open at a preset or adjustable opening pressure. The use of VPS in iNPH patients is often indicated if a patient responds well to a tap test, during which CSF is drawn out through lumbar puncture to transiently simulate shunt placement [9]. While the first ETV procedure was performed in 1922 by Dr. Walter Dandy via an open craniectomy approach, VPS remained the first-line surgical treatment for iNPH throughout most of the 20th century until advances in modern endoscopes led to a reemergence of ETV as a viable alternative [10-12]. In 1999, Mitchell and Matthew reported the first use of ETV to treat iNPH [13].

Treatment with both VPS and ETV has proven to be effective at reversing the symptoms of iNPH with reasonable complication and survival rates [14-18]. Despite this, opinions remain mixed as to whether or not ETV should be used over the current VPS standard [19]. Some studies have found that ETV leads to greater clinical improvement and lower complication rates than VPS [20-22]. Others, however, have found that VPS is associated with better neurological outcomes and sustained resolution of symptoms compared to ETV [23-25]. Given the continued debate over the comparative efficacy of these two approaches and the contradictory findings reported in prior studies, we aimed to investigate this question further using a large health record database.

## Materials and methods

Study design

This study is a retrospective propensity score-matched analysis using TriNetX, a multi-institutional health record database. TriNetX houses de-identified electronic medical records from over 150 million patients globally, spanning dozens of healthcare organizations and academic medical institutions. The database is updated continuously and includes information regarding patient demographics, diagnoses, medications, and outcomes. Within the database, patients are isolated via diagnostic and procedural codes, including those from the International Classification of Diseases (ICD), Current Procedural Terminology (CPT), and Systematized Nomenclature of Medicine Clinical Terms (SNOMED). This study utilized the TriNetX United States Collaborative Network (USCN), a network contained within the TriNetX database that contains information on over 120 million patients from 70 academic institutions across the United States. Since the TriNetX database is federated, an Institutional Review Board approval for this study has been waived.

Patient selection and outcome analysis

Patient Cohorts

The USCN was queried for patients aged 50 years and older who received a diagnosis of iNPH. Given that iNPH tends to be diagnosed in older age, a lower bound of 50 years was chosen to capture a greater number of patients for analysis and exclude only rare cases in younger individuals. These patients were then separated into two cohorts based on their subsequent treatment with either an ETV or VPS procedure. Specifically, patients in the ETV cohort were included if they had been treated with an ETV following iNPH diagnosis but were never treated with a VPS or other ventricular shunt type. Similarly, patients in the VPS cohort were included if they had been treated with a VPS following iNPH diagnosis, but never treated with an ETV or other ventricular shunt type. A complete list of the codes used to define each cohort is included in Appendix 1.

Propensity Score Matching

The two cohorts were then propensity score-matched based on the following characteristics: age, sex, race, disorders of lipoprotein metabolism and other lipidemias, malaise and fatigue, diabetes mellitus, overweight and obesity status, acute kidney failure and chronic kidney disease, aphagia and dysphagia, ischemic heart disease, hypertensive diseases, atrial fibrillation and flutter, heart failure, congenital hydrocephalus and other congenital brain malformations, peripheral vascular disease, fibrosis or cirrhosis of the liver, chronic lower respiratory disease, somnolence, stupor and coma, symptoms and signs concerning food and fluid intake, nicotine and alcohol dependence or abuse, and antithrombotic drugs including aspirin, warfarin, apixaban, rivaroxaban, and clopidogrel. Prior to matching, there were 6,496 patients in the VPS cohort and 125 patients in the ETV cohort. Matching is performed directly by the TriNetX online platform and resulted in 118 matched patients in each cohort for subsequent outcome analysis. A complete list of the codes used to define propensity score matching characteristics is included in Appendix 2.

Outcomes and Statistical Analysis

The outcomes compared across the two cohorts included death, subdural hematoma, seizures, falls, urinary incontinence, emergency department visits, CT of the head, and MRI of the brain. The codes used to determine CT, MRI, and emergency department visits do not specify the reason for the encounter, so any instance of these events was included for these outcomes. The time window for outcome analysis was any time within 10 years following the index event, defined as either the ETV or VPS procedure after iNPH diagnosis. Statistics are performed directly by the TriNetX online platform with chi-square analysis used for categorical variables. If an outcome occurs in one to 10 patients in a given cohort, TriNetX will round up to 10 patients to use for statistical analysis to protect patient confidentiality. The significance level was set at a p-value < 0.05. A complete list of the codes used to define each outcome is included in Appendix 3.

## Results

After propensity score matching, the difference in demographic characteristics between the VPS and ETV cohorts, including age at the time of surgery, sex, and race, was not significant. Data on the differences in demographic characteristics between the two cohorts after matching are listed in Table 1.

**Table 1:** Matched cohort demographics. VPS: ventriculoperitoneal shunt; ETV: endoscopic third ventriculostomy; SD: standard deviation; Std. mean diff.: standardized mean difference; * indicates statistical significance at p < 0.05.

Characteristic	VPS cohort	ETV cohort	p-value	Std. mean diff.
					Age at the time of surgery (years, mean ± SD)	65.9 ± 8.3	67.2 ± 8.9	0.2506	0.1499
Male	57.63%	52.54%	0.4323	0.1024					
					White	72.88%	71.19%	0.7718	0.0378
Black or African American	12.71%	8.48%	0.2902	0.1380					
					Asian	8.48%	8.48%	1	0

After matching, cohorts did not significantly differ in comorbidities or antithrombotic drug use, with the exception of fibrosis and cirrhosis of the liver, which was significantly higher in the VPS cohort (p = 0.0012). Differences in comorbidities and antithrombotic drug use between the two cohorts after matching are listed in Table 2.

**Table 2:** Matched cohort comorbidities and antithrombotic drug use. VPS: ventriculoperitoneal shunt; ETV: endoscopic third ventriculostomy; Std. mean diff.: standardized mean difference; * indicates statistical significance at p < 0.05.

Comorbidity/antithrombotic drug		VPS cohort	ETV cohort	p-value	Std. mean diff.
						Disorders of lipoprotein metabolism and other lipidemias		40.68%	41.53%	0.8947	0.0172
Malaise and fatigue		11.86%	18.64%	0.1475	0.1894						
						Diabetes mellitus		15.25%	19.49%	0.3903	0.1120
Overweight, obesity and other hyperalimentation		16.10%	18.64%	0.6063	0.0671						
						Acute kidney failure and chronic kidney disease		12.71%	16.10%	0.4584	0.0966
Aphagia and dysphagia		10.17%	12.71%	0.5395	0.0799						
						Ischemic heart diseases		16.10%	15.25%	0.8579	0.0233
Hypertensive diseases		47.46%	52.54%	0.4347	0.1018						
						Atrial fibrillation and flutter		11.02%	9.32%	0.6667	0.0561
Heart failure		8.48%	8.48%	1	0						
						Congenital hydrocephalus		11.86%	16.10%	0.3480	0.1224
Other congenital malformations of the brain		8.48%	8.48%	1	0						
						Other peripheral vascular diseases		8.48%	8.48%	1	0
Fibrosis and cirrhosis of the liver		8.48%	0%	0.0012*	0.4303						
						Chronic lower respiratory diseases		19.49%	15.25%	0.3903	0.1120
Somnolence, stupor, and coma		8.48%	8.48%	1	0						
						Symptoms and signs concerning food and fluid intake		8.48%	8.48%	1	0
Personal history of nicotine dependence		12.71%	14.41%	0.7037	0.0495						
						Nicotine dependence		11.02%	9.32%	0.6667	0.0561
Alcohol dependence		8.48%	8.48%	1	0						
						Alcohol abuse		8.48%	8.48%	1	0
Aspirin		24.58%	32.20%	0.1938	0.1698						
						Warfarin		8.48%	8.48%	1	0
Apixaban		8.48%	8.48%	1	0						
						Rivaroxaban		8.48%	8.48%	1	0
Clopidogrel		8.48%	8.48%	1	0						

The analysis of clinical outcomes between patients in the VPS and ETV cohorts showed no significant differences in the risk of death (risk ratio (RR) = 0.941, p = 0.8510), subdural hematoma (RR = 1.081, p = 0.8520), seizures (RR = 1.040, p = 0.9272), falls (RR = 1.818, p = 0.0829), or urinary incontinence (RR = 0.667, p = 0.2902). However, patients in the VPS cohort were significantly more likely to have a subsequent emergency department visit (RR = 1.536, p = 0.0333) and were more likely to receive a CT scan of the head (RR = 1.438, p = 0.0001). In contrast, patients in the ETV cohort were significantly more likely to receive an MRI of the brain (RR = 0.271, p < 0.0001). The results of the outcome analysis are presented in Table 3.

**Table 3:** Outcome differences by treatment for iNPH. VPS: ventriculoperitoneal shunt; ETV: endoscopic third ventriculostomy; iNPH: idiopathic normal pressure hydrocephalus; CI: confidence interval; * indicates statistical significance at p < 0.05.

Outcome	Cohort	Risk	Risk difference (95% CI)	Risk ratio (95% CI)	Odds ratio (95% CI)	p-value
							Death	VPS	13.68%	-0.86% (-9.77%, 8.10%)	0.941 (0.500, 1.772)	0.932 (0.446, 1.946)	0.8510
ETV	14.53%												
Subdural hematoma	VPS	9.65%	0.72% (-6.85%, 8.29%)	1.081 (0.478, 2.443)	1.089 (0.443, 2.677)	0.8520
	ETV	8.93%				
Seizures	VPS	9.90%	0.38% (-7.71%, 8.47%)	1.040 (0.452, 2.391)	1.044 (0.415, 2.626)	0.9272
	ETV	9.52%				
Falls	VPS	16.95%	7.63% (-0.94%, 16.19%)	1.818 (0.912, 3.625)	1.985 (0.905, 4.353)	0.0829
	ETV	9.32%				
Urinary incontinence	VPS	8.48%	-4.24% (-12.07%, 3.60%)	0.667 (0.312, 1.423)	0.636 (0.273, 1.479)	0.2902
	ETV	12.71%				
Emergency department visit	VPS	36.44%	12.71% (1.12%, 24.30%)	1.536 (1.028, 2.295)	1.843 (1.046, 3.246)	0.0333*
	ETV	23.73%				
CT head	VPS	77.97%	23.73% (12.04%, 35.42%)	1.438 (1.187, 1.741)	2.986 (1.695, 5.260)	0.0001*
	ETV	54.24%				
MRI brain	VPS	16.10%	-43.22% (-54.29%, -32.15%)	0.271 (0.175, 0.421)	0.132 (0.071, 0.243)	<0.0001*
	ETV	59.32%				

## Discussion

The results of our analysis showed that compared to patients treated with a VPS, those treated with an ETV were significantly less likely to visit the emergency department and receive a CT scan of the head, and significantly more likely to receive an MRI of the brain. In contrast, all other clinical outcomes were comparable between the two cohorts. Collectively, these findings imply that few differences in outcomes exist between the two treatment approaches.

The observed differences in imaging modalities between the two cohorts likely arise from the fundamental characteristics of each procedure. As VPS patients have an implanted device, CT offers an efficient way to quickly observe and evaluate shunt functionality. Likewise, ETV patients would be more likely to undergo MRI as this modality offers better visualization of soft tissue structures and would aid in the evaluation of the stoma made on the floor of the third ventricle during the procedure.

Regarding emergency department visits, while it is possible that the increased risk observed in the VPS group could result from complications arising from the VPS implantation procedure or subsequent device failure, this cannot be confidently asserted given that the reason for the visits was unknown. However, if an increased risk of complications in VPS patients is the underlying explanation, this would align with prior studies that reported higher complication rates in these patients. For example, one study compared the outcomes of 25 iNPH patients treated with ETV to compiled outcomes from 14 shunt studies, reporting that none of the ETV patients experienced subdural collections, while VPS patients experienced subdural effusions or hematomas at an average rate of 16.3% [20]. Moreover, they reported a neurological improvement rate of 72% for the ETV patients, compared to 66% for the shunted patients. From these findings, the authors concluded that since ETV provides similar clinical improvement to shunting with lower complication rates, it should be the first choice of treatment for iNPH.

In contrast to these reports, other studies have found that VPS is associated with more favorable outcomes, which reinforces the argument that the higher proportion of emergency department visits observed in the VPS group in our study may not be related to complications associated with the procedure. For example, one study of 652 patients treated with ETV and 12,845 treated with VPS found that treatment with ETV was associated with a significantly higher mortality rate of 3.2%, compared to just 0.5% for treatment with VPS [15]. Additionally, ETV was associated with a short-term complication rate of 17.9%, versus just 11.8% for VPS. Another study that examined self-reported outcomes in iNPH patients found greater quality of life improvements in shunted patients compared to those who were treated with an ETV procedure [26].

While these prior studies assert more favorable outcomes with either VPS or ETV, the results of our study largely show that outcomes are comparable between treatments, as cohorts did not differ significantly in mortality, subdural hematoma, seizures, falls, or urinary incontinence. This conclusion aligns with that of a review published in 2019 that analyzed 2,461 patients across 33 studies and found that outcomes did not differ significantly by treatment type [27]. Taken together, although more work is needed to confirm the comparative efficacy of VPS and ETV, our findings suggest either surgical approach is a viable option for the treatment of patients with iNPH.

This study has several limitations. First, given that the methodology relies on billing codes, it is possible that outcomes could be incorrectly detected and included in the results because of common codes used to define multiple outcomes. Another potential confounder is selection bias resulting from the substantial difference in initial cohort sizes (6,496 in the VPS group compared to just 125 in the ETV group). Given that only a small number of patients from the VPS cohort were matched to those in the ETV cohort, this sample may not be representative of VPS patients as a whole. Regarding the comparison of clinical outcomes, morbidity, and functional status could not be readily tested, which limits the conclusions on comparative treatment efficacy that can be drawn. Moreover, the specific indication for CT or MRI and emergency department visits could not be determined. Thus, instances of these outcomes could be unrelated to a patient's ETV, VPS, or iNPH status. Lastly, as with any database study, a degree of misidentification and miscoding is possible.

## Conclusions

The results of our study suggest that patients with iNPH experience similar clinical outcomes whether treated surgically with VPS or ETV. Observed differences in imaging modalities can be explained by the nature of each procedure and although a greater proportion of patients in the VPS cohort had a subsequent visit to the emergency department, this cannot be confidently attributed to complications inherent to the procedure. Although our study benefits from the ability to pull patient data on a national scale, it is limited in its ability to elucidate more granular data on patient morbidity and functional outcomes. Our findings align with some previous studies that have concluded no difference in complications between treatment with VPS or ETV, although others have reported higher rates of complications in one of the two procedures. Ultimately, more evidence is needed to definitively establish if one approach provides superior symptom resolution for iNPH patients.

## Appendices

Appendix 1: Codes used to define cohorts

ETV Cohort

Patients must have the code:

- G91.2 (Idiopathic) normal pressure hydrocephalus (≥50 years)

And at least one of the following codes (after the occurrence of the above code):

- 1014234 Ventriculocisternostomy, third ventricle - 62180 Ventriculocisternostomy (Torkildsen type operation) - 00160ZB Bypass Cerebral Ventricle to Cerebral Cisterns, Open Approach - 00163ZB Bypass Cerebral Ventricle to Cerebral Cisterns, Percutaneous Approach - 00164ZB Bypass Cerebral Ventricle to Cerebral Cisterns, Percutaneous Endoscopic Approach - 00163JB Bypass Cerebral Ventricle to Cerebral Cisterns with Synthetic Substitute, Percutaneous Approach - 00160JB Bypass Cerebral Ventricle to Cerebral Cisterns with Synthetic Substitute, Open Approach - 001607B Bypass Cerebral Ventricle to Cerebral Cisterns with Autologous Tissue Substitute, Open Approach - 001637B Bypass Cerebral Ventricle to Cerebral Cisterns with Autologous Tissue Substitute, Percutaneous Approach - 00160KB Bypass Cerebral Ventricle to Cerebral Cisterns with Nonautologous Tissue Substitute, Open Approach - 00163KB Bypass Cerebral Ventricle to Cerebral Cisterns with Nonautologous Tissue Substitute, Percutaneous Approach - 00164JB Bypass Cerebral Ventricle to Cerebral Cisterns with Synthetic Substitute, Percutaneous Endoscopic Approach - 00164KB Bypass Cerebral Ventricle to Cerebral Cisterns with Nonautologous Tissue Substitute, Percutaneous Endoscopic Approach

And cannot have any of the following codes:

- 02.42 Replacement of ventricular shunt - 1009325 Creation of shunt - 1009331 Creation of shunt - 0016070 Bypass Cerebral Ventricle to Nasopharynx with Autologous Tissue Substitute, Open Approach - 0016071 Bypass Cerebral Ventricle to Mastoid Sinus with Autologous Tissue Substitute, Open Approach - 0016072 Bypass Cerebral Ventricle to Atrium with Autologous Tissue Substitute, Open Approach - 0016073 Bypass Cerebral Ventricle to Blood Vessel with Autologous Tissue Substitute, Open Approach - 0016074 Bypass Cerebral Ventricle to Pleural Cavity with Autologous Tissue Substitute, Open Approach - 0016075 Bypass Cerebral Ventricle to Intestine with Autologous Tissue Substitute, Open Approach - 0016076 Bypass Cerebral Ventricle to Peritoneal Cavity with Autologous Tissue Substitute, Open Approach - 0016077 Bypass Cerebral Ventricle to Urinary Tract with Autologous Tissue Substitute, Open Approach - 0016078 Bypass Cerebral Ventricle to Bone Marrow with Autologous Tissue Substitute, Open Approach - 00160J0 Bypass Cerebral Ventricle to Nasopharynx with Synthetic Substitute, Open Approach - 00160J1 Bypass Cerebral Ventricle to Mastoid Sinus with Synthetic Substitute, Open Approach - 00160J2 Bypass Cerebral Ventricle to Atrium with Synthetic Substitute, Open Approach - 00160J3 Bypass Cerebral Ventricle to Blood Vessel with Synthetic Substitute, Open Approach - 00160J4 Bypass Cerebral Ventricle to Pleural Cavity with Synthetic Substitute, Open Approach - 00160J5 Bypass Cerebral Ventricle to Intestine with Synthetic Substitute, Open Approach - 00160J6 Bypass Cerebral Ventricle to Peritoneal Cavity with Synthetic Substitute, Open Approach - 00160J7 Bypass Cerebral Ventricle to Urinary Tract with Synthetic Substitute, Open Approach - 00160J8 Bypass Cerebral Ventricle to Bone Marrow with Synthetic Substitute, Open Approach - 00160JA Bypass Cerebral Ventricle to Subgaleal Space with Synthetic Substitute, Open Approach - 00160K0 Bypass Cerebral Ventricle to Nasopharynx with Nonautologous Tissue Substitute, Open Approach - 00160K1 Bypass Cerebral Ventricle to Mastoid Sinus with Nonautologous Tissue Substitute, Open Approach - 00160K2 Bypass Cerebral Ventricle to Atrium with Nonautologous Tissue Substitute, Open Approach - 00160K3 Bypass Cerebral Ventricle to Blood Vessel with Nonautologous Tissue Substitute, Open Approach - 00160K4 Bypass Cerebral Ventricle to Pleural Cavity with Nonautologous Tissue Substitute, Open Approach - 00160K5 Bypass Cerebral Ventricle to Intestine with Nonautologous Tissue Substitute, Open Approach - 00160K6 Bypass Cerebral Ventricle to Peritoneal Cavity with Nonautologous Tissue Substitute, Open Approach - 00160K7 Bypass Cerebral Ventricle to Urinary Tract with Nonautologous Tissue Substitute, Open Approach - 00160K8 Bypass Cerebral Ventricle to Bone Marrow with Nonautologous Tissue Substitute, Open Approach - 00160KA Bypass Cerebral Ventricle to Subgaleal Space with Nonautologous Tissue Substitute, Open Approach - 0016370 Bypass Cerebral Ventricle to Nasopharynx with Autologous Tissue Substitute, Percutaneous Approach - 0016371 Bypass Cerebral Ventricle to Mastoid Sinus with Autologous Tissue Substitute, Percutaneous Approach - 0016372 Bypass Cerebral Ventricle to Atrium with Autologous Tissue Substitute, Percutaneous Approach - 0016373 Bypass Cerebral Ventricle to Blood Vessel with Autologous Tissue Substitute, Percutaneous Approach - 0016374 Bypass Cerebral Ventricle to Pleural Cavity with Autologous Tissue Substitute, Percutaneous Approach - 0016375 Bypass Cerebral Ventricle to Intestine with Autologous Tissue Substitute, Percutaneous Approach - 0016376 Bypass Cerebral Ventricle to Peritoneal Cavity with Autologous Tissue Substitute, Percutaneous Approach - 0016377 Bypass Cerebral Ventricle to Urinary Tract with Autologous Tissue Substitute, Percutaneous Approach - 0016378 Bypass Cerebral Ventricle to Bone Marrow with Autologous Tissue Substitute, Percutaneous Approach - 00163J0 Bypass Cerebral Ventricle to Nasopharynx with Synthetic Substitute, Percutaneous Approach - 00163J1 Bypass Cerebral Ventricle to Mastoid Sinus with Synthetic Substitute, Percutaneous Approach - 00163J2 Bypass Cerebral Ventricle to Atrium with Synthetic Substitute, Percutaneous Approach - 00163J3 Bypass Cerebral Ventricle to Blood Vessel with Synthetic Substitute, Percutaneous Approach - 00163J4 Bypass Cerebral Ventricle to Pleural Cavity with Synthetic Substitute, Percutaneous Approach - 00163J5 Bypass Cerebral Ventricle to Intestine with Synthetic Substitute, Percutaneous Approach - 00163J6 Bypass Cerebral Ventricle to Peritoneal Cavity with Synthetic Substitute, Percutaneous Approach - 00163J7 Bypass Cerebral Ventricle to Urinary Tract with Synthetic Substitute, Percutaneous Approach - 00163J8 Bypass Cerebral Ventricle to Bone Marrow with Synthetic Substitute, Percutaneous Approach - 00163JA Bypass Cerebral Ventricle to Subgaleal Space with Synthetic Substitute, Percutaneous Approach - 00163K0 Bypass Cerebral Ventricle to Nasopharynx with Nonautologous Tissue Substitute, Percutaneous Approach - 00163K1 Bypass Cerebral Ventricle to Mastoid Sinus with Nonautologous Tissue Substitute, Percutaneous Approach - 00163K2 Bypass Cerebral Ventricle to Atrium with Nonautologous Tissue Substitute, Percutaneous Approach - 00163K3 Bypass Cerebral Ventricle to Blood Vessel with Nonautologous Tissue Substitute, Percutaneous Approach - 00163K4 Bypass Cerebral Ventricle to Pleural Cavity with Nonautologous Tissue Substitute, Percutaneous Approach - 00163K5 Bypass Cerebral Ventricle to Intestine with Nonautologous Tissue Substitute, Percutaneous Approach - 00163K6 Bypass Cerebral Ventricle to Peritoneal Cavity with Nonautologous Tissue Substitute, Percutaneous Approach - 00163K7 Bypass Cerebral Ventricle to Urinary Tract with Nonautologous Tissue Substitute, Percutaneous Approach - 00163K8 Bypass Cerebral Ventricle to Bone Marrow with Nonautologous Tissue Substitute, Percutaneous Approach - 0016474 Bypass Cerebral Ventricle to Pleural Cavity with Autologous Tissue Substitute, Percutaneous Endoscopic Approach - 0016475 Bypass Cerebral Ventricle to Intestine with Autologous Tissue Substitute, Percutaneous Endoscopic Approach - 0016476 Bypass Cerebral Ventricle to Peritoneal Cavity with Autologous Tissue Substitute, Percutaneous Endoscopic Approach - 00164J2 Bypass Cerebral Ventricle to Atrium with Synthetic Substitute, Percutaneous Endoscopic Approach - 00164J4 Bypass Cerebral Ventricle to Pleural Cavity with Synthetic Substitute, Percutaneous Endoscopic Approach - 00164J5 Bypass Cerebral Ventricle to Intestine with Synthetic Substitute, Percutaneous Endoscopic Approach - 00164J6 Bypass Cerebral Ventricle to Peritoneal Cavity with Synthetic Substitute, Percutaneous Endoscopic Approach - 00164J7 Bypass Cerebral Ventricle to Urinary Tract with Synthetic Substitute, Percutaneous Endoscopic Approach - 00164JA Bypass Cerebral Ventricle to Subgaleal Space with Synthetic Substitute, Percutaneous Endoscopic Approach - 00164K1 Bypass Cerebral Ventricle to Mastoid Sinus with Nonautologous Tissue Substitute, Percutaneous Endoscopic Approach - 00164K6 Bypass Cerebral Ventricle to Peritoneal Cavity with Nonautologous Tissue Substitute, Percutaneous Endoscopic Approach - 1009521 Creation of shunt, lumbar, subarachnoid-peritoneal, -pleural, other

VPS Cohort

Patients must have the code:

- G91.2 (Idiopathic) normal pressure hydrocephalus (≥50 years)

And at least one of the following codes (after the occurrence of the above code):

- 62223 Creation of shunt; ventriculo-peritoneal, -pleural, other terminus - 00164K6 Bypass Cerebral Ventricle to Peritoneal Cavity with Nonautologous Tissue Substitute, Percutaneous Endoscopic Approach - 00164J6 Bypass Cerebral Ventricle to Peritoneal Cavity with Synthetic Substitute, Percutaneous Endoscopic Approach - 0016476 Bypass Cerebral Ventricle to Peritoneal Cavity with Autologous Tissue Substitute, Percutaneous Endoscopic Approach - 00163K6 Bypass Cerebral Ventricle to Peritoneal Cavity with Nonautologous Tissue Substitute, Percutaneous Approach - 00163J6 Bypass Cerebral Ventricle to Peritoneal Cavity with Synthetic Substitute, Percutaneous Approach - 0016376 Bypass Cerebral Ventricle to Peritoneal Cavity with Autologous Tissue Substitute, Percutaneous Approach - 00160K6 Bypass Cerebral Ventricle to Peritoneal Cavity with Nonautologous Tissue Substitute, Open Approach - 00160J6 Bypass Cerebral Ventricle to Peritoneal Cavity with Synthetic Substitute, Open Approach - 0016076 Bypass Cerebral Ventricle to Peritoneal Cavity with Autologous Tissue Substitute, Open Approach

And cannot have any of the following codes:

- 02.42 Replacement of ventricular shunt - 1014234 Ventriculocisternostomy, third ventricle - 62180 Ventriculocisternostomy (Torkildsen type operation) - 00160ZB Bypass Cerebral Ventricle to Cerebral Cisterns, Open Approach - 00163ZB Bypass Cerebral Ventricle to Cerebral Cisterns, Percutaneous Approach - 00164ZB Bypass Cerebral Ventricle to Cerebral Cisterns, Percutaneous Endoscopic Approach - 00163KB Bypass Cerebral Ventricle to Cerebral Cisterns with Nonautologous Tissue Substitute, Percutaneous Approach - 00164KB Bypass Cerebral Ventricle to Cerebral Cisterns with Nonautologous Tissue Substitute, Percutaneous Endoscopic Approach - 00164JB Bypass Cerebral Ventricle to Cerebral Cisterns with Synthetic Substitute, Percutaneous Endoscopic Approach - 00163JB Bypass Cerebral Ventricle to Cerebral Cisterns with Synthetic Substitute, Percutaneous Approach - 001607B Bypass Cerebral Ventricle to Cerebral Cisterns with Autologous Tissue Substitute, Open Approach - 001637B Bypass Cerebral Ventricle to Cerebral Cisterns with Autologous Tissue Substitute, Percutaneous Approach - 00160KB Bypass Cerebral Ventricle to Cerebral Cisterns with Nonautologous Tissue Substitute, Open Approach - 00160JB Bypass Cerebral Ventricle to Cerebral Cisterns with Synthetic Substitute, Open Approach - 00160J5 Bypass Cerebral Ventricle to Intestine with Synthetic Substitute, Open Approach - 00163J5 Bypass Cerebral Ventricle to Intestine with Synthetic Substitute, Percutaneous Approach - 00163J2 Bypass Cerebral Ventricle to Atrium with Synthetic Substitute, Percutaneous Approach - 00160J2 Bypass Cerebral Ventricle to Atrium with Synthetic Substitute, Open Approach - 00160J0 Bypass Cerebral Ventricle to Nasopharynx with Synthetic Substitute, Open Approach - 00163J0 Bypass Cerebral Ventricle to Nasopharynx with Synthetic Substitute, Percutaneous Approach - 0016075 Bypass Cerebral Ventricle to Intestine with Autologous Tissue Substitute, Open Approach - 0016375 Bypass Cerebral Ventricle to Intestine with Autologous Tissue Substitute, Percutaneous Approach - 00160K5 Bypass Cerebral Ventricle to Intestine with Nonautologous Tissue Substitute, Open Approach - 00163K5 Bypass Cerebral Ventricle to Intestine with Nonautologous Tissue Substitute, Percutaneous Approach - 00160J8 Bypass Cerebral Ventricle to Bone Marrow with Synthetic Substitute, Open Approach - 00163J8 Bypass Cerebral Ventricle to Bone Marrow with Synthetic Substitute, Percutaneous Approach - 00160J4 Bypass Cerebral Ventricle to Pleural Cavity with Synthetic Substitute, Open Approach - 00163J4 Bypass Cerebral Ventricle to Pleural Cavity with Synthetic Substitute, Percutaneous Approach - 0016072 Bypass Cerebral Ventricle to Atrium with Autologous Tissue Substitute, Open Approach - 0016372 Bypass Cerebral Ventricle to Atrium with Autologous Tissue Substitute, Percutaneous Approach - 00160K2 Bypass Cerebral Ventricle to Atrium with Nonautologous Tissue Substitute, Open Approach - 00163K2 Bypass Cerebral Ventricle to Atrium with Nonautologous Tissue Substitute, Percutaneous Approach - 00160J3 Bypass Cerebral Ventricle to Blood Vessel with Synthetic Substitute, Open Approach - 00163J3 Bypass Cerebral Ventricle to Blood Vessel with Synthetic Substitute, Percutaneous Approach - 00160J1 Bypass Cerebral Ventricle to Mastoid Sinus with Synthetic Substitute, Open Approach - 00163J1 Bypass Cerebral Ventricle to Mastoid Sinus with Synthetic Substitute, Percutaneous Approach - 0016070 Bypass Cerebral Ventricle to Nasopharynx with Autologous Tissue Substitute, Open Approach - 0016370 Bypass Cerebral Ventricle to Nasopharynx with Autologous Tissue Substitute, Percutaneous Approach - 00160K0 Bypass Cerebral Ventricle to Nasopharynx with Nonautologous Tissue Substitute, Open Approach - 00163K0 Bypass Cerebral Ventricle to Nasopharynx with Nonautologous Tissue Substitute, Percutaneous Approach - 00160J7 Bypass Cerebral Ventricle to Urinary Tract with Synthetic Substitute, Open Approach - 00163J7 Bypass Cerebral Ventricle to Urinary Tract with Synthetic Substitute, Percutaneous Approach - 00164J2 Bypass Cerebral Ventricle to Atrium with Synthetic Substitute, Percutaneous Endoscopic Approach - 00164J5 Bypass Cerebral Ventricle to Intestine with Synthetic Substitute, Percutaneous Endoscopic Approach - 00160JA Bypass Cerebral Ventricle to Subgaleal Space with Synthetic Substitute, Open Approach - 00163JA Bypass Cerebral Ventricle to Subgaleal Space with Synthetic Substitute, Percutaneous Approach - 0016078 Bypass Cerebral Ventricle to Bone Marrow with Autologous Tissue Substitute, Open Approach - 0016378 Bypass Cerebral Ventricle to Bone Marrow with Autologous Tissue Substitute, Percutaneous Approach - 00160K8 Bypass Cerebral Ventricle to Bone Marrow with Nonautologous Tissue Substitute, Open Approach - 00163K8 Bypass Cerebral Ventricle to Bone Marrow with Nonautologous Tissue Substitute, Percutaneous Approach - 0016074 Bypass Cerebral Ventricle to Pleural Cavity with Autologous Tissue Substitute, Open Approach - 0016374 Bypass Cerebral Ventricle to Pleural Cavity with Autologous Tissue Substitute, Percutaneous Approach - 00160K4 Bypass Cerebral Ventricle to Pleural Cavity with Nonautologous Tissue Substitute, Open Approach - 00163K4 Bypass Cerebral Ventricle to Pleural Cavity with Nonautologous Tissue Substitute, Percutaneous Approach - 0016073 Bypass Cerebral Ventricle to Blood Vessel with Autologous Tissue Substitute, Open Approach - 0016373 Bypass Cerebral Ventricle to Blood Vessel with Autologous Tissue Substitute, Percutaneous Approach - 00160K3 Bypass Cerebral Ventricle to Blood Vessel with Nonautologous Tissue Substitute, Open Approach - 00163K3 Bypass Cerebral Ventricle to Blood Vessel with Nonautologous Tissue Substitute, Percutaneous Approach - 0016071 Bypass Cerebral Ventricle to Mastoid Sinus with Autologous Tissue Substitute, Open Approach - 0016371 Bypass Cerebral Ventricle to Mastoid Sinus with Autologous Tissue Substitute, Percutaneous Approach - 00160K1 Bypass Cerebral Ventricle to Mastoid Sinus with Nonautologous Tissue Substitute, Open Approach - 00163K1 Bypass Cerebral Ventricle to Mastoid Sinus with Nonautologous Tissue Substitute, Percutaneous Approach - 0016077 Bypass Cerebral Ventricle to Urinary Tract with Autologous Tissue Substitute, Open Approach - 0016377 Bypass Cerebral Ventricle to Urinary Tract with Autologous Tissue Substitute, Percutaneous Approach - 00160K7 Bypass Cerebral Ventricle to Urinary Tract with Nonautologous Tissue Substitute, Open Approach - 00163K7 Bypass Cerebral Ventricle to Urinary Tract with Nonautologous Tissue Substitute, Percutaneous Approach - 0016475 Bypass Cerebral Ventricle to Intestine with Autologous Tissue Substitute, Percutaneous Endoscopic Approach - 00164J4 Bypass Cerebral Ventricle to Pleural Cavity with Synthetic Substitute, Percutaneous Endoscopic Approach - 00160KA Bypass Cerebral Ventricle to Subgaleal Space with Nonautologous Tissue Substitute, Open Approach - 00164JA Bypass Cerebral Ventricle to Subgaleal Space with Synthetic Substitute, Percutaneous Endoscopic Approach - 00164J7 Bypass Cerebral Ventricle to Urinary Tract with Synthetic Substitute, Percutaneous Endoscopic Approach - 00164K1 Bypass Cerebral Ventricle to Mastoid Sinus with Nonautologous Tissue Substitute, Percutaneous Endoscopic Approach - 0016474 Bypass Cerebral Ventricle to Pleural Cavity with Autologous Tissue Substitute, Percutaneous Endoscopic Approach

Appendix 2: Codes used to define propensity score matching characteristics

- AI: Age at index - M: Male - F: Female - 2106-3 White - 2054-5 Black or African American - 2028-9 Asian - E78 Disorders of lipoprotein metabolism and other lipidemias - R53 Malaise and fatigue - E08-E13 Diabetes mellitus - E65-E68 Overweight, obesity and other hyperalimentation - N17-N19 Acute kidney failure and chronic kidney disease - R13 Aphagia and dysphagia - I20-I25 Ischemic heart diseases - I10-I16 Hypertensive diseases - I48 Atrial fibrillation and flutter - I50 Heart failure - Q03 Congenital hydrocephalus - Q04 Other congenital malformations of brain - I73 Other peripheral vascular diseases - K74 Fibrosis and cirrhosis of liver - J40-J47 Chronic lower respiratory diseases - R40 Somnolence, stupor and coma - R63 Symptoms and signs concerning food and fluid intake - Z87.891 Personal history of nicotine dependence - F17 Nicotine dependence - F10.2 Alcohol dependence - F10.1 Alcohol abuse - 1191 Aspirin - 11289 Warfarin - 1364430 Apixaban - 1114195 Rivaroxaban - 32968 Clopidogrel

Appendix 3: Codes used to define outcomes

Outcome: Death

Patients must have the following code:

- Deceased

Outcome: Subdural Hematoma

Patients must have the following code:

- I62.00 Nontraumatic subdural hemorrhage, unspecified

Outcome: Seizures

Patients must have at least one of the following codes:

- R56 Convulsions, not elsewhere classified - G40 Epilepsy and recurrent seizures

Outcome: Falls

Patients must have the following code:

- W19 Unspecified fall

Outcome: Urinary Incontinence

Patients must have the following code:

- R32 Unspecified urinary incontinence

Outcome: Emergency Department Visit

Patients must have the following code:

- 1013711 Emergency Department Services

Outcome: CT Scan of the Head

Patients must have at least one of the following codes:

- 24726-2 CT Head WO and W contrast IV - 30799-1 CT Head WO contrast - 70460 Computed tomography, head or brain; with contrast material(s) - 70450 Computed tomography, head or brain; without contrast material - 303653007 CT of head - 429858000 CT of head and neck - 408754009 CT of entire head - 582101000119108 CT angiography of head with contrast - BW28 Anatomical Regions/Computerized Tomography (CT Scan)/Head - BW29 Anatomical Regions/Computerized Tomography (CT Scan)/Head And Neck - 24729-6 CT perfusion Head WO and W contrast IV - 42377-2 CT perfusion Head W Xe-133 IH+WO and W contrast IV

Outcome: MRI of the Brain

Patients must have at least one of the following codes:

- 816077007 MRI of brain - 29567006 MRI of brain and brain stem - B030 Central Nervous System/Magnetic Resonance Imaging (MRI)/Brain - 1010326 Magnetic resonance (e.g., proton) imaging, brain (including brain stem)
